# Gender Disparities and Burnout Among Emergency Physicians: A Systematic Review by the World Academic Council of Emergency Medicine–Female Leadership Academy for Medical Excellence

**DOI:** 10.5811/westjem.29331

**Published:** 2024-12-31

**Authors:** Suman Thakur, Vivek Chauhan, Sagar Galwankar, Fatimah Lateef, Pia Daniel, Zeynep Cakir, Katia M. Lugo, Samjhana Basnet, Busra Bildik, Siham Azahaf, Sevilay Vural, Busra H. Difyeli, Lisa Moreno-Walton

**Affiliations:** * Indira Gandhi Medical College & Hospital, Department of Emergency Medicine, Shimla, India; † Indira Gandhi Medical College & Hospital, Department of Medicine, Shimla, India; ‡ Sarasota Memorial Hospital, Florida State University College of Medicine Emergency Medicine Residency Program, Department of Emergency Medicine, Sarasota, Florida; § Singapore General Hospital, Department of Emergency Medicine, Singapore; ∥ Downstate Health Sciences University, Department of Emergency Medicine, Brooklyn, New York; ¶ Ataturk University, Department of Emergency Medicine, Erzurum, Türkiye; # HCA/USF Morsani COM: GME Oak Hill Hospital, Department of Emergency Medicine, Brooksville, Florida; ** Dhulikhel Hospital Kathmandu University Hospital, Department of General Practice and Emergency Medicine, Kavre, Nepal; †† Karabuk University, Faculty of Medicine, Department of Emergency Medicine, Karabuk, Türkiye; ‡‡ Amsterdam University Medical Centers, Department of Internal Medicine, Amsterdam, Netherlands; §§ Yozgat Bozok University, Department of Emergency Medicine, Azizli, Türkiye; ∥∥ Almus State Hospital, Department of Emergency Medicine, Almus/Tokat, Türkiye; ¶¶ Louisiana State University Health Sciences Center, Section of Emergency Medicine, New Orleans, Louisiana

## Abstract

**Background:**

The Female Leadership Academy for Medical Excellence, members of the World Academic Council of Emergency Medicine, conducted this systematic review, which explores gender disparities in burnout among emergency physicians (EP) using the Maslach Burnout Inventory-Human Services Survey (MBI-HSS). Burnout is a critical issue in healthcare, particularly in emergency medicine where high stress and demanding work environments prevail.

**Methods:**

Following PRISMA guidelines, we searched PubMed and Epistemonikos for studies using MBI-HSS to measure burnout in EPs. Inclusion criteria encompassed peer-reviewed, English-language articles reporting burnout by sex. Data extraction focused on proportions of burnout and its subcomponents, mean scores, and odds ratios, with quality assessed using Joanna Briggs Institute criteria.

**Results:**

We included 18 studies spanning 26,939 EPs from 10 countries. While overall burnout rates did not significantly differ between the sexes, the proportion of female EPs with high emotional exhaustion (EE) (69%) and low sense of personal accomplishment (PA) (45%) were significantly higher compared to males with high EE in 57% and low PA in 29%, respectively (*P* < 0.001 for both). Proportion with high depersonalization (DP) score was 44% in both male and female EPs. Mean scores revealed females experiencing higher mean EE (26.8 ± 15.7) scores vs males (25.4 ± 15.9) *P* < 0.001. Males had mean DP scores (8.6 ± 8.0) and mean PA scores (26.6 ± 12.7) compared to females with lower mean DP scores (7.4 ± 7.2) and higher PA scores (27.7 ± 11.9), respectively *P* < 0.001 for both. Odds ratios indicated varying risks, predominantly higher EE odds among females, varying from 0.72 to 2.3.

**Conclusion:**

This review underscores gender-specific manifestations of burnout among emergency physicians, with females more susceptible to emotional exhaustion and lower sense of personal accomplishment. Standardized reporting methods are crucial for future meta-analyses to refine gender-specific interventions combating burnout in emergency medicine. Targeted strategies addressing distinct manifestations of burnout are imperative to support the well-being and retention of EPs, fostering sustainable healthcare delivery.

## INTRODUCTION

The term “burnout,” introduced by Freudenberger in 1974, refers to job-related dissatisfaction primarily caused by work-related stress.^1^ The most widely validated tool for measuring burnout among physicians is the Maslach Burnout Inventory-Human Services Survey (MBI-HSS) 22-item tool.^2^ The MBI-HSS measures burnout in three subcomponents: emotional exhaustion (EE); depersonalization (DP); and personal accomplishment (PA).^2^ Burnout is suggested by a high score in EE and DP, and a low score on PA.^2^

There is a palpable gender gap in academic emergency medicine (EM) where female emergency physicians (EP) are less likely to hold major leadership positions, more likely to spend a greater percentage of time in clinical and teaching activities, publish less in peer-reviewed journals, and are less likely to achieve senior academic ranks in their medical schools.^3^ Even after adjusting for factors such as race, region, rank, years of experience, clinical hours, core faculty status, administrative roles, board certification, and fellowship training, the mean (±SD) salary of women was found to be $19,418 (±$3,736) less than that of men (*P* < 0.001).^4^ This gender disparity can negatively impact the retention of female EPs and predispose them to higher burnout.

Although there are systematic reviews that have described burnout among EPs, none have focused on the gender gap in burnout among EPs.^5^ ^–^ ^7^ Therefore, the Female Leadership Academy for Medical Excellence (FLAME) members of the World Academic Council of Emergency Medicine performed a systematic review to describe the gender disparity in burnout among EPs at a global level. To the best of our knowledge, this is the first systematic review focusing on gender disparity in burnout among EPs as measured by the validated MBI tool.

## METHODS

We performed a systematic review following the PRISMA methods^8^ using the protocol published in PROSPERO (CRD42024558794).

### Search Strategy

We searched two open access databases, PubMed and Epistemonikos on June 30, 2024, for peer -reviewed articles on burnout and emergency physicians. We operationalized different permutations of each keyword as follows:

Burnout: “Maslach burnout inventory” OR MBI OR burnout OR burn-out OR “burned out” OR depersonalization OR “emotional exhaustion” OR “compassion fatigue”

Emergency Physician: “emergency physician*” OR “emergency doctor*” OR “EM physician*” OR “EM doctor*” OR “emergency resident*” OR “EM resident*” OR “emergency consultant*” OR “EM consultant*” OR “emergency faculty*” OR “EM faculty*” OR “emergency professor*” OR “EM professor*” OR “emergency attending*” OR “EM attending*”

We applied the field “All fields” for searching on PubMed and “Title and Abstracts” for searching the same combination of keywords in Epistemonikos.

### Screening and Eligibility

We applied a series of inclusion and exclusion criteria. Articles were included if they were 1) written in English, 2) published in a peer-reviewed journal, 3) original articles, and 4) applied any version of the MBI-HSS to measure burnout. They were excluded if they 1) did not describe the results separately by sex, 2) did not include EPs in their study, or 3) were a systematic review.

### Extraction and Analysis

Extraction was performed by two investigators independently. The following information was extracted: study characteristics (first author, year of publication, country, number of participants that responded); characteristics of participants (mean age, proportion of males and females); and outcome data (proportion of high burnout in males and females, proportion of males and females with high EE, high DP and low PA, mean scores in males and females for EE, DP or PA and odds of burnout or its subcomponents—EE, EP, or PA—in female EPs.

### Study Quality

We used the Joanna Briggs Institute’s critical appraisal checklist for evaluation of the quality of the prevalence studies.^9^ The tool assessed quality using nine questions. A score of 1 was assigned for a “Yes” as an answer, and a score of 0 was assigned for an answer that was “No,” “Unclear,” or “Not Applicable.” The scores were graded as low, moderate or high if the total score was ≤ 4, 5–7, and ≥ 8, respectively. The quality assessment was performed independently by two investigators, and any disagreement was settled by discussion.

## RESULTS

### Literature Search

Our initial search resulted in 331 articles in PubMed and 13 in Epistemonikos, which were imported into EndNote reference management software (Clarivate Analytics, Philadelphia, PA). Of these 344 articles, nine were found to be duplicates, leaving a total of 335 articles for the screening and eligibility stages (Figure). Of the 335 articles screened, we excluded 226 that did not meet the inclusion criteria, leaving us with 109 articles for retrieval. We reviewed these 109 full texts for eligibility, resulting in the exclusion of the following:

• 3 articles that were systematic reviews
• 7 articles that were not peer-reviewed original articles
• 5 articles that did not include emergency physicians
• 41 articles that did not use the Maslach Burnout Inventory for measuring burnout
• 35 articles that did not report their data by sex,

**Figure.**
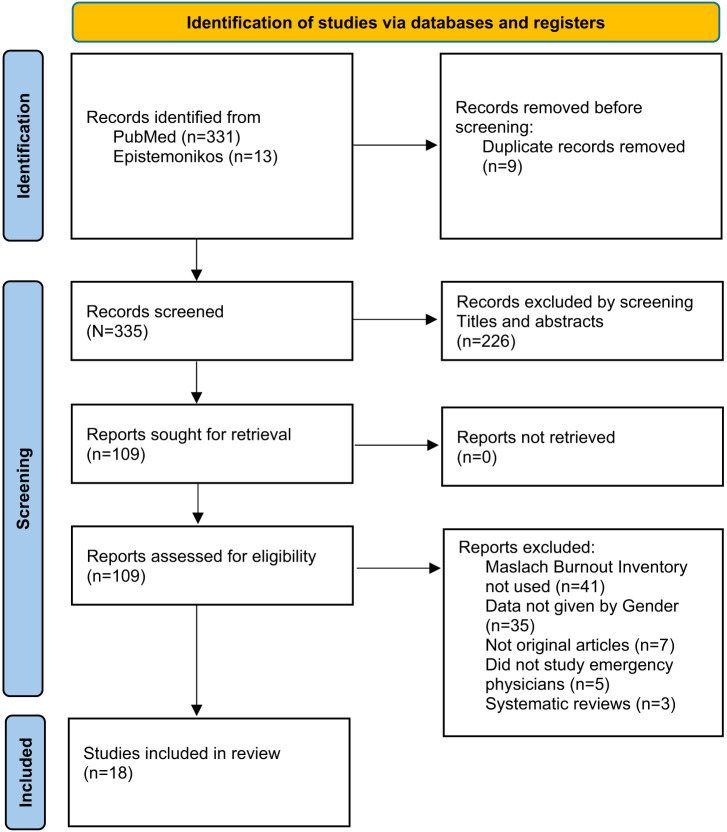
Prisma flow diagram.

This left a total of 18 articles for the final review. The process of screening and selecting studies is shown in the PRISMA flow diagram (Figure).

## STUDY CHARACTERISTICS

We included 18 studies from 10 different countries in the final analysis (Table 1). The total number of EPs studied in these 18 studies was 26,939, including 8,864 (33%) female EPs, resulting in a male-to-female ratio of 2:1. Fifteen of these studies used the 22-item MBI-HSS tool for measuring burnout, while two studies used the two-item tool based on the MBI-HSS, and one included the nine-item MBI tool (Table 1). All were multicentric studies except for one, which was a single-center study. Included were EPs of both sexes in all studies except for one, which included only female EPs. All included studies were conducted in the last 10 years (2014 to present), except for one study conducted in 1996. Six studies were scored as high quality, while the remaining 12 were moderate quality studies. The response rates varied from 30–94.1%.

**Table 1.** Characteristics of the included studies.

First author	Year	Country	Response	Quality	MBI tool	Total N=26,939	Males n=18,075 (67%)	Females n=8,864 (33%)
Batur A^18^	2023	Türkiye	NA	7	22 item	389	223	166
De Wit K^19^	2020	Canada	40%	7	2 item	467	240	227
Elhadi M^20^	2021	Libya	77%	7	9 item	154	82	72
Erdur B^21^	2015	Türkiye	85%	7	22 item	174	138	36
Feeks C^22^	2020	US	31%	7	22 item	139	49	90
Goldberg R^23^	1996	US	NA	7	22 item	1,272	945	327
Iyer S^24^	2022	Tanzania	77%	7	22 item	29	20	9
Jalili M^25^	2013	Iran	88%	8	22 item	164	150	14
Kimo TJ	2014	US	75%	9	22 item	218	129	89
Liu R^27^	2020	Canada	30%	7	22 item	65	38	27
Lovell LP^28^	2022	Barbados	63%	8	22 item	111	49	62
Lu DW^29^	2022	US	94.1%	7	2 item	7,466	4,768	2,698
Mercuri M^30^	2021	Canada	58%	8	22 item	416	214	202
Morikawa M^31^	2023	Japan	81.9%	9	22 item	267	214	53
Salmoirago BE^32^	2016	US	32.7%	7	2 item	138	100	38
Sarwar S^33^	2023	Pakistan	NA	6	22 item	150	66	84
Soltanifar A^34^	2018	Iran	71%	8	22 item	77	0	77
Yan S^35^	2021	China	NA	8	22 item	15,243	10,650	4,593

*MBI*, Maslach Burnout Inventory.

## OUTCOME ANALYSIS

### Burnout calculated by Maslach Burnout Inventory

Seven studies reported the burnout proportions separately in male and female EPs (Table 2). The studies that included the 22-item MBI-HSS tool had a total of 1,181 male and 542 female EPs, with an average pooled proportion of high burnout at 58.7% and 58.3%, respectively (*P* = 0.8). Two studies used the two-item MBI, including a total of 4,868 male and 2,736 female EPs, with an average pooled proportion of high burnout at 27% and 34%, respectively (*P* < 0.001).

**Table 2.** Proportion of male and female emergency physicians with high levels of burnout.

Author	MBI	Males (n=1,181)	Burnout (%) n=695 (58.8%)	Females (n=542)	Burnout (%) n=317 (58.4%)	*P-*value (Fisher exact)
22-item MBI tool						
Feeks C^22^	22 item	49	6 (13%)	90	35 (39%)	0.8
Goldberg R^23^	22 item	945	567 (60%)	327	203 (62%)	
Iyer^24^	22 item	20	13 (65%)	9	6 (69%)	
Kimo TJ	22 item	129	88 (68%)	89	54 (61%)	
Liu R^27^	22 item	38	21 (55%)	27	19 (69%)	
2-item MBI tool						
		(n=4,868)	n=1,502 (31.4%)	(n=2,736)	n=903 (33%)	
Lu DW^29^	2 item	4,768	1,478 (31%)	2,698	890 (33%)	0.05
Salmoirago BE^32^	2 item	100	24 (24%)	38	13 (35%)	

*MBI,* Maslach Burnout Inventory.

### Subcomponents of MBI

Seven studies reported individual components of the MBI-HSS, specifically the proportion of participants with high EE, high DP, and low PA separately for male and female EPs (Tables 3 and 4). Four of these studies reported the proportion of participants with subcomponents suggestive of burnout (Table 3), and three of these studies reported the mean and standard deviations of the MBI subcomponents (Table 4). Female EPs had proportionately higher EE and lower PA than male EPs (*P* < 0.001). The DP levels were similar among male and female EPs (Table 3). The combined mean EE score was higher in female EPs (*P* < 0.001), while the combined PA score was higher and the DP score was lower in female EPs compared to male EPs (*P* < 0.001) (Table 4). Four studies presented their results in the form of odds or relative risk (Table 5), and three of these showed higher odds among females of burnout while one had lower odds. Three of these studies reported only one subcomponent of MBI-HSS (ie, EE) (Table 5).

**Table 3. tab3:** Proportion of participants with high levels of emotional exhaustion, high depersonalization, and low sense of personal accomplishment, the individual components of the Maslach Burnout Inventory, among male and female emergency physicians.

Author	Male (n=371)	High EE	Low PA	High DP	Female (n=399)	High EE	Low PA	High DP
Batur A^18^	223	135 (61%)	69 (31%)	-	166	119 (72%)	83 (50%)	-
Elhadi M^20^	82	51 (62%)	21 (25%)	39 (47%)	72	53 (74%)	13 (18%)	35 (49%)
Sarwar S^33^	66	26 (39%)	18 (27%)	27 (41%)	84	40 (48%)	22 (26%)	31 (37%)
Soltanifar A^34^	0	-	-	-	77	65 (85%)	62 (81%)	37 (48%)
Total	371	212 (57%)	108 (29%)	66 (44%)	399	277 (69%)	180 (45%)	103 (44%)
*P-*value (Fisher exact test)						<0.001	<0.001	0.77

*EE*, emotional exhaustion; *PA*, personal accomplishment; *DP*, depersonalization.

**Table 4. tab4:** Mean (standard deviation) values of subcomponents of the Maslach Burnout Inventory among male and female emergency physicians.

Author	Male (n=10,837)	High EE	Low PA	Low DP	Female (n=4,691)	High EE	Low PA	High DP
Lovell LP^28^	49	29.4 (11.7)	45.5 (8)	11.8 (5.7)	62	32.5 (12.1)	43.4 (6.8)	13 (6.7)
Yan S^35^	10,650	25.4 (16.1)	26.5 (12.8)	8.5 (8.1)	4,593	26.8 (15.7)	27.6 (11.9)	7.4 (7.2)
Erdur B^21^	138	24.6 (6.0)	29.9 (3.9)	10.7 (4.1)	36	24.1 (6.7)	30.0 (3.4)	11.0 (3.2)
Combined	10,837	25.4 (15.9)	26.6 (12.7)	8.6 (8.0)	4,691	26.8 (15.7)	27.7 (11.9)	7.4 (7.2)
Unpaired *t*-test *P-*value			<0.001	<0.001		<0.001		

*EE*, emotional exhaustion; *PA*, personal accomplishment; *DP*, depersonalization.

**Table 5.** Odds or relative risk of burnout or its subcomponents by gender among emergency physicians.

Authors	Males	Females	Reported parameter	Value
De Wit K^19^	240	227	Odds of burnout in males	0.54 (0.22–1.35)
Jalili M^25^	150	14	Relative risk emotional exhaustion in females	1.05
Mercuri M^30^	214	202	Odds of emotional exhaustion in females	2.32
Morikawa M^31^	214	53	Odds of emotional exhaustion in females	0.72 (0.28–1.79)

## DISCUSSION

The stressful environment of EM is a known contributor to the negative impacts of burnout.^10^ Burnout can be the result of good-intentioned physicians who strive for perfection at work.^10^ Emergency physicians become frustrated when their work environment falls short of supporting well-meaning goals, leading to EP burnout.^10^

Moral injury is now recognized as a significant factor contributing to burnout among EPs. They often face challenging decisions such as prioritizing care in life-or-death situations, dealing with resource limitations, and frequently witnessing suffering and death. The emotional toll of moral injury can lead to symptoms of depression, anxiety and stress disorders, which are closely linked to burnout. Female EPs, in addition, face sex-based discrimination, bias, unequal treatment, and fewer opportunities for career advancement.^3^ They are more burdened with balancing professional and family responsibilities. Females are expected to display more empathy and provide emotional support to patients and colleagues, which can increase emotional labor and moral injury if they are unable to meet these expectations.

The MBI assesses the severity of the three primary symptoms of burnout: exhaustion; depersonalization; and lack of personal accomplishment. Developed in the 1970s, it has become the gold standard for measuring burnout across various professions and industries. The MBI-HSS is specifically designed for use in human services professions such as social work, counseling, and healthcare.^2^ A burnout survey of 7,288 US physicians from all specialties, using the MBI-HSS tool, showed that high burnout was reported by 38% of US physicians and that burnout is more common among physicians than other US workers.^11^ Among all specialties, EM had the highest burnout rates, with over 60% of EPs reporting high burnout levels.^11^ Emergency medicine is a frontline specialty, and several factors contribute to high burnout in EPs, including night shifts, sleep disorders, job-related strain, fear of making mistakes, and workplace violence.^12^

In recent years, more female physicians have entered the workforce, leading to increased data availability for studying sex differences in burnout symptoms. In some cultures, female patients preferentially ask for female EPs to attend to them in the ED.^13^ A recently conducted systematic review of US physicians found that women physicians have a higher likelihood of experiencing burnout compared to male physicians, particularly with respect to the EE dimension of burnout.^14^ Studies focusing on EPs corroborate this finding, indicating that female EPs are at higher risk of burnout compared to male EPs. Additionally, females have higher attrition rates compared to their male counterparts.^3^ ^,^ ^4^ ^,^ ^15^ Factors cited as contributing to the discrepancy in burnout include greater levels of work-family conflict, greater tendencies to emotionally invest in patients/work, and greater discrimination in salaries and promotions by female physicians.^14^

In contrast to the above studies, findings from a systematic review of 16,016 physicians from the Eastern Mediterranean region indicated no significant difference in burnout rates between male and female physicians.^16^ However, none of the studies in Doraiswamy’s systematic review were designed to compare differences by sex, limiting the interpretations of the findings.^15^ Another systematic review that included 109,628 physicians concluded that inconsistencies in definitions and assessment methods for burnout across studies prevented a reliable determination of the association between burnout and the sex of the physician.^17^

These reviews highlight variability in findings across different regions and contexts, suggesting that the relationship between burnout and sex may vary depending on factors such as cultural norms, healthcare system characteristics, and study methodologies. Therefore, while some studies may indicate a gender disparity in burnout, others may not find such differences, emphasizing the need for nuanced interpretation and context-specific understanding of burnout in healthcare professions.

We conducted this systematic review to address inconsistent data on the gender gap in burnout among EPs. Rotenstein et al have highlighted in their review the inconsistencies in the definitions and assessment methods of burnout; therefore, we focused specifically on studies that used the MBI-HSS tool for assessing burnout.^17^ Despite using a common assessment tool, the included studies employed various methods for reporting burnout scores. Of the 18 studies included in our review,^18^ ^–^ ^35^ seven reported burnout as the percentage of participants with high burnout; four reported the percentage of participants with high individual components of burnout (EE, DP, PA) but not overall burnout; three reported the mean scores for EE, DP, and PA; and four reported odds ratios and relative risks for EE (Tables 2 [Table tab3] [Table tab4]–5). This approach allowed us to comprehensively examine and compare the gender disparities in burnout among EPs across different studies.

We collated data from studies reporting burnout as a percentage of the population having high burnout and found that of 1,181 male EPs and 542 female EPs, high burnout was reported in 58.8% and 58.4%, respectively, which indicates nearly equal rates of burnout between male and female EPs^22^ ^–^ ^24^ ^,^ ^26^ ^,^ ^27^ (Table 2). The two-item tool, known for its brevity and ease of administration, can effectively identify at-risk EM residents showing early signs of burnout.^36^ This tool uses two questions from the MBI-HSS and enables consistent, widespread, and longitudinal monitoring of burnout among EM residents at local, regional, and national levels.^36^ In our systematic review, we included two studies that used the two-item tool to measure burnout among 4,778 male and 1,502 female EPs. The reported burnout rates were 31% among male EPs and 33% among female EPs (*P* = 0.05).^29^ ^,^ ^32^ This slight difference suggests a trend toward higher burnout among female EPs, as indicated by these specific studies using the abbreviated MBI tool.

Four additional studies included in our systematic review, totalling 317 male and 399 female EPs, reported individual components of burnout (EE, DP, PA) as percentages of participants with high or low scores (Table 3).^18^ ^,^ ^20^ ^,^ ^33^ ^,^ ^34^ These studies found that the proportion of female EPs with high EE and low PA was significantly higher compared to male EPs (*P* < 0.001). However, levels of DP were comparable between both genders (*P* = 0.77). This indicates that female EPs may experience greater EE and lower PA, highlighting potential areas of concern for gender-specific burnout interventions in emergency medicine.

Lastly, among the remaining studies that reported odds ratios or relative risks for burnout, 3 of 4 studies indicated a higher risk of burnout among female EPs, while one study showed a higher risk among male EPs (Table 5).^19^ ^,^ ^25^ ^,^ ^30^ ^,^ ^31^ Specifically, female EPs were found to have a higher risk for the EE component of burnout compared to male EPs, as suggested by these findings. This underscores the gender disparity in burnout risk within the EM profession, emphasizing the need for targeted interventions to mitigate these disparities and support the well-being of all EPs.

In our systematic review, we encountered challenges in performing a meta-analysis due to the variability in how authors reported their findings using the MBI tool to measure burnout among EPs. Specifically, there were limitations stemming from the limited number of studies that reported results separately for male and female EPs, as well as the diversity in how parameters of burnout were reported across these studies. The variation in reporting included differences in the following:

• Whether burnout was reported as overall scores or individual components (EE, DP, PA)
• The specific metrics used to define high burnout
• The methods used to analyze and present data (percentages, means, odds ratios, etc).

This variability makes it challenging to aggregate data across studies for a meta-analysis, which typically requires a consistent approach to data reporting and statistical measures. As a result, while our review provides valuable insights into the gender disparities in burnout among EPs, the heterogeneity in reporting prevents a quantitative synthesis of the findings.

Moving forward, standardizing the reporting of MBI-HSS results and burnout parameters in future studies would facilitate more robust meta-analytical approaches to further elucidate the gender gap in burnout among EPs and inform targeted interventions to mitigate burnout in this critical healthcare specialty.

As per the originators of the MBI-HSS the pre-2016 versions of the tool used arbitrary classifications of high burnout, dividing the normative population into tertiles labelled as high, moderate, and low burnout. This approach was later acknowledged as a mistake, leading to the removal of these cutoff classifications from all versions of the MBI-HSS starting with the fourth edition in 2016. This change allowed researchers to view burnout as a continuum within the context of specific populations.^37^

In our systematic review, despite including 14 studies conducted after 2016, only three studies reported the mean (±SD) scores of individual components of burnout separately for male and female EPs.^21^ ^,^ ^28^ ^,^ ^35^ Notably, these studies highlighted significant differences: females exhibited significantly higher EE scores, while males showed significantly higher DP and lower PA scores (*P* < 0.001).^21^ ^,^ ^28^ ^,^ ^35^ It is important to note that the study by Yan et al in China included a large cohort of 15,243 participants, which may skew the overall findings toward this study’s results.^35^

In most other studies included in our review, researchers derived cutoffs by combining results from individual components (EE, DP, PA), which could explain why overall burnout scores appeared equivocal across these studies (Table 2). This variability in reporting underscores the ongoing challenge of harmonizing burnout measurements across different studies and emphasizes the need for standardized reporting practices to facilitate clearer comparisons and meta-analyses in future research.

Twelve of the articles included in our study were published in 2020 or later, and five studies were conducted during the peak of the COVID-19 pandemic in 2020–2021. It is likely that the pandemic caused unusual fatigue and burnout, resulting in different burnout levels among males and females across different regions.

## LIMITATIONS

This systematic review encountered several limitations. One major challenge was the variability in how studies reported burnout, even when using the MBI-HSS tool. Some studies focused on overall burnout, while others reported individual components such as EE, DP and PA. Additionally, studies employed different metrics (percentages, means, or odds ratios), making it difficult to aggregate findings for a meta-analysis. The limited number of studies reporting gender-specific data further constrained our ability to make definitive conclusions about the gender gap in burnout among EPs. Moreover, some studies used outdated versions of the MBI-HSS, which relied on arbitrary cutoffs for high burnout, affecting the accuracy of burnout classification. Finally, several studies were conducted during the COVID-19 pandemic, a period marked by increased fatigue and burnout, potentially skewing the findings and limiting their generalizability beyond that time frame.

## CONCLUSION

This review highlights that the science of examining physician burnout is complex and influenced by a wide range of factors. No two studies are perfectly comparable, even when using similar assessment tools, such as the Maslach Burnout Inventory-Human Services Survey. Variables like the type of work, workload, acuity of tasks, job satisfaction, hours worked, frequency of rapid decision-making, critical thinking demands, work-life balance, competing interests, cultural beliefs, and societal norms—such as power distance and hierarchy—all impact burnout differently.

Despite the findings that female emergency physicians report higher emotional exhaustion and lower sense of personal accomplishment than their male counterparts, it is essential for societies and countries to delve deeper into this issue, tailoring studies to their specific contexts and cultures. This review underscores the need for gender-specific strategies to combat burnout among EPs. While both men and women experience significant burnout, the nature of their burnout differs, necessitating targeted interventions to support the well-being of all EPs.

### Recommendations for Research

1. **Uniform Reporting of Results:** Future studies on burnout among EPs should uniformly report results, including the mean (±SD) for the individual components of the MBI-HSS tool— EE, DP and PA—for both male and female EPs. This standardized approach will facilitate a clearer understanding of the gender gap in burnout across different settings and populations.
2. **Further Research Focus:** There is a critical need for further research to delve into the underlying reasons behind the disparities in EE, DP, and PA between male and female EPs. Understanding these factors is essential for developing targeted interventions that address the specific needs of each gender, thereby effectively mitigating burnout.
3. **Move Away from Arbitrary Cutoff Scores:** The practice of using arbitrary cutoff scores (high, moderate, low burnout) to categorize burnout levels should be abandoned. This approach, discouraged by the originators of the MBI-HSS tool, does not accurately capture the nuanced experiences of burnout and may lead to misleading conclusions.
4. **Holistic Assessment of Burnout:** Rather than focusing solely on overall burnout scores, future studies should emphasize the detailed assessment of EE, DP, and PA. This holistic approach provides a more comprehensive understanding of burnout dynamics among EPs and allows for targeted interventions based on specific components of burnout.

### Call to Action by FLAME

Based on the observations regarding burnout among women in EM and female EPs, we are proposing the following measures:

1. **Increased Awareness and Education:**
  ○ Enhance awareness and recognition of burnout within the EM community.
  ○ Incorporate burnout-related sessions into EM residency training and core curriculum.
2. **Proactive Faculty and Leadership:**
  ○ Faculty and EM leadership should closely monitor all staff, including both female and male EPs, for signs of work-related stress, cognitive overload, and other relevant commitments.
  ○ Emphasize addressing burnout as a work-related issue rather than a gender-related one, especially when there is no confirmed evidence of gender-specific causes.
3. **Open Discussions on Burnout:**
  ○ Foster an open, non-judgmental dialogue about burnout during departmental peer-review sessions.
  ○ Ensure that staff at all levels can share their experiences while maintaining psychological safety.
  ○ Address burnout openly to prevent it from becoming a “silent crisis.”
4. **Psychological Wellness Initiatives:**
  ○ Leadership and management should implement psychological wellness initiatives, such as “Joy @ Work,” iTHRIVE initiatives, and wellness grants.
5. **Peer-Support Committees:**
  ○ Establish interprofessional peer-support committees or teams.
  ○ Encourage staff to discuss burnout with peers, who may be more approachable, and share best practices for managing burnout.
